# Supplementary material for: Shared Treg dysregulation underlies psoriasis vulgaris and atopic dermatitis through the PHF19-PRC2 epigenetic axis
Source: Front Immunol. 2026 Aug 20;17:1932788. doi: 10.3389/fimmu.2026.1932788 (PMC13538097; doi:10.3389/fimmu.2026.1932788)
Supplement: Supplementary file 3 [file DataSheet3.zip › Additional files/Additional file 2/31.NTPCR.forest.pdf]

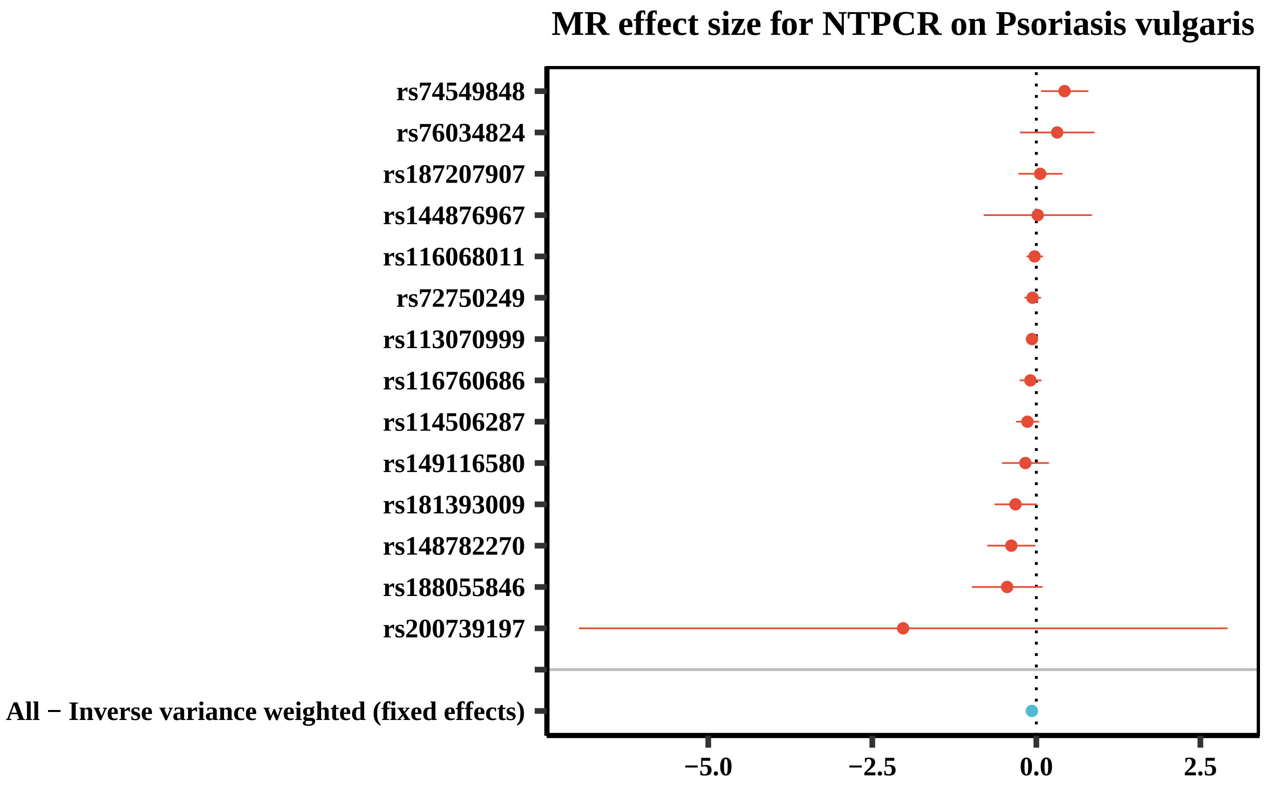

MR effect size for NTPCR on Psoriasis vulgaris
rs74549848
rs76034824
rs187207907
rs144876967
rs116068011
rs72750249
rs113070999
rs116760686
rs114506287
rs149116580
rs181393009
rs148782270
rs188055846
rs200739197
All − Inverse variance weighted (fixed effects)
−5.0
−2.5
0.0
2.5
